# Comparison of Transfemoral versus Transsubclavian/Transaxillary access for transcatheter aortic valve replacement: A systematic review and meta-analysis

**DOI:** 10.1016/j.ijcha.2022.101156

**Published:** 2022-12-01

**Authors:** Waiel Abusnina, Akshay Machanahalli Balakrishna, Mahmoud Ismayl, Azka Latif, Mostafa Reda Mostafa, Ahmad Al-abdouh, Muhammad Junaid Ahsan, Qais Radaideh, Toufik M. Haddad, Andrew M. Goldsweig, Itsik Ben-Dor, Mamas A. Mamas, Khagendra Dahal

**Affiliations:** a Division of Cardiology, Creighton University School of Medicine, NE, USA; b Rochester Regional Health/Unity Hospital, Rochester, NY, USA; c Department of Medicine, University of Kentucky, Lexington, KY, USA; d Division of Cardiovascular Medicine, Iowa Heart Center, Des Moines, IA, USA; e Division of Cardiovascular Medicine, University of Nebraska Medical Center, Omaha, NE, USA; f Section of Interventional Cardiology, MedStar Washington Hospital Center, Washington DC, USA; g Keele Cardiovascular Research Group, Keele University, UK

**Keywords:** TAVR, TAVI, Access site, Subclavian access, Axillary access, Femoral access, AKI, Acute Kidney Injury, AS, Aortic Stenosis, CI, Confidence Interval, MI, Myocardial Infarction, RR, Risk Ratio, TAVR, Transcatheter Aortic Valve Replacement, TF, Transfemoral, TSc, Transsubclavian, TAx, Transaxillary, TC, Transcarotid

## Abstract

Femoral access is the gold standard for transcatheter aortic valve replacement (TAVR). Safe alternative access, that represents about 15 % of TAVR cases, remains important for patients without adequate transfemoral access. We aimed to perform a systematic review and *meta*-analysis of studies comparing transfemoral (TF) access versus transsubclavian or transaxillary (TSc/TAx) access in patients undergoing TAVR. We searched PubMed, Cochrane CENTRAL Register, EMBASE, Web of Science, Google Scholar and ClinicalTrials.gov (inception through May 24, 2022) for studies comparing (TF) to (TSc/TAx) access for TAVR. A total of 21 studies with 75,995 unique patients who underwent TAVR (73,203 transfemoral and 2,792 TSc/TAx) were included in the analysis. There was no difference in the risk of in-hospital and 30-day all-cause mortality between the two groups (RR 0.64, 95 % CI 0.36–1.13, P = 0.12) and (RR 0.95, 95 % CI 0.64–1.41, P = 0.81), while 1-year mortality was significantly lower in the TF TAVR group (RR 0.79, 95 % CI 0.67–0.93, P = 0.005). No significant differences in major bleeding (RR 0.82, 95 % CI 0.65–1.03, P = 0.09), major vascular complications (RR 1.14, 95 % CI 0.75–1.72, P = 0.53), and stroke (RR 0.66, 95 % CI 0.42–1.02, P = 0.06) were observed. In patients undergoing TAVR, TF access is associated with significantly lower 1-year mortality compared to TSc/TAx access without differences in major bleeding, major vascular complications and stroke. While TF is the preferred approach for TAVR, TSc/TAx is a safe alternative approach. Future studies should confirm these findings, preferably in a randomized setting.

## 1 Introduction

Aortic valve replacement for symptomatic severe aortic stenosis (AS) has class I indication in both the current guidelines.[1], [2] Transcatheter aortic valve replacement (TAVR) has been approved for aortic valve replacement in high-, intermediate- or low-risk patients with symptomatic severe AS [3], [4], [5], [6] becoming a predominant therapy for the treatment of severe AS, exceeding surgical aortic valve replacement in the US since 2019.[7] As delivery systems have evolved, corresponding sheath sizes have also become smaller to facilitate greater rates of transfemoral (TF) TAVR. While TF access remains the preferred access route for TAVR,[8] 10–15 % of cases are unsuitable for TF access.[9] TF route allows a fully-percutaneous TAVR under conscious sedation/local anesthesia. Careful procedural planning by CT scan and accurate choice of the proper site for vascular puncture are keys for procedural success. Analysis of CT scan images will help to identify potential challenges such as tortuosity, presence of aneurysms, thrombotic appositions, or aortic arch calcifications. All these anatomic features are potential sources of complications when large catheters are inserted and, therefore, can be considered as relative contraindications for a transfemoral approach. When TF access is contraindicated, an alternate access like trans subclavian or transaxillary or trans carotid or transaortic can be considered. Due to unfavorable outcomes associated with transapical and transaortic access,[10], [11] other alternative access routes have been developed including transsubclavian (TSc) and transaxillary (TAx).[12], [13], [14] While preferences for alternative access TAVR approaches vary and depend on operator preference, institutional experience and patient anatomy, alternative access site choice is critical.

Important TAVR outcomes include access-related complications like pseudoaneurysm or bleeding. [15], [16] Comparing outcomes between non-TF access versus TF access is clinically important in defining outcomes associated with alternative access. While evidence shows advantages and disadvantages of each access routes, due to lack of head-to-head randomized comparator trials, appropriate access choice remains a debatable/controversial issue. We, therefore, aimed to review all studies comparing TF to TSc/TAx accesses in regards to their safety and efficacy endpoints through a systematic review and *meta*-analysis.

## 2 Methods:

### 2.1 Data sources and search strategy

A meta-analysis was performed according to the Preferred Reporting Items for Systematic Reviews on meta-Analyses (PRISMA) 2015 guidelines. [17] Two reviewers (WA, MI) independently identified the relevant studies by an electronic search of the PubMed, EMBASE, Cochrane Central Register of Controlled Trials, and ClinicalTrials.gov databases (from inception to May 2022). Reference lists of the retrieved studies were also screened further for relevant studies. The following search terms and key words were used: “aortic stenosis” and “transcatheter aortic valve replacement” or “TAVR” or “TAVI” and “subclavian artery access” or “axillary artery access” or “femoral artery access”. The *meta*-analysis was registered in the PROSPERO (International Prospective Register of Systematic Reviews) Registry, under PROSPERO CRD42022340351.

### 2.2 Study selection

Two reviewers (WA, MI) independently assessed studies’ eligibility based on titles, abstracts, and full-text reports. Discrepancies in study selection were discussed and resolved with a third investigator (KD). Eligible studies had to compare between transsubclavian and/or transaxillary vs transfemoral access for TAVR, and present clinical outcomes data of interest. Exclusion criteria were: (a) lack of any clinical outcome data, (b) single arm studies, (c) duplicate publications, (d) reviews, editorials, letters, and non-human studies. Only studies published in the English language were included in this *meta*-analysis.

### 2.3 Data extraction and quality assessment

**Supplementary figure 1:**
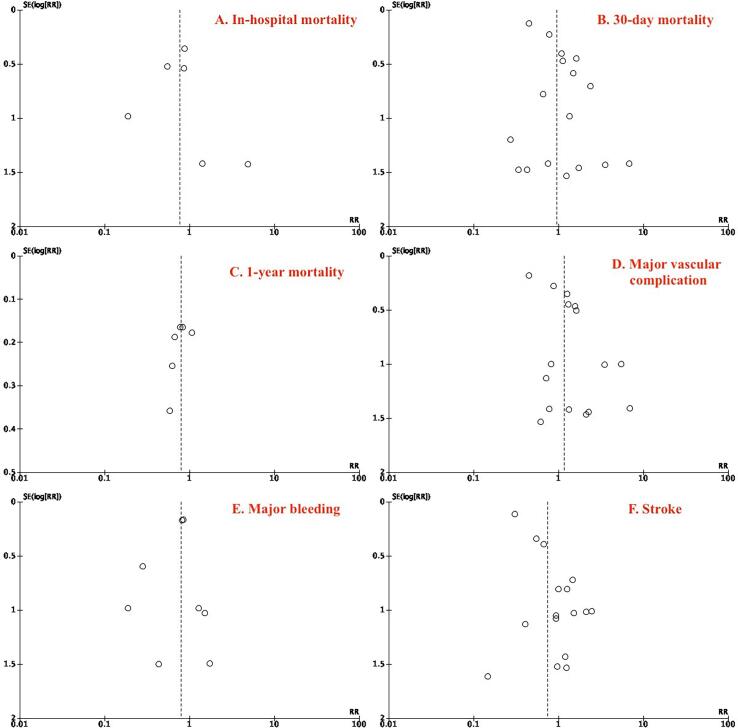


Two investigators (WA, AMB) independently extracted data (baseline characteristics, outcomes and number of events) using a standardized data abstraction form. Funnel plots for the outcomes were used to assess for publication bias when data were available for at least three studies **(Supplemental** Fig. 1, Fig. 2**)**. The studies' methodological quality was assessed systematically using the Newcastle-Ottawa Scale for observational studies **(Supplemental** Table 2**)**, and disagreements were resolved by a third author (KD).

**Fig. 1:**
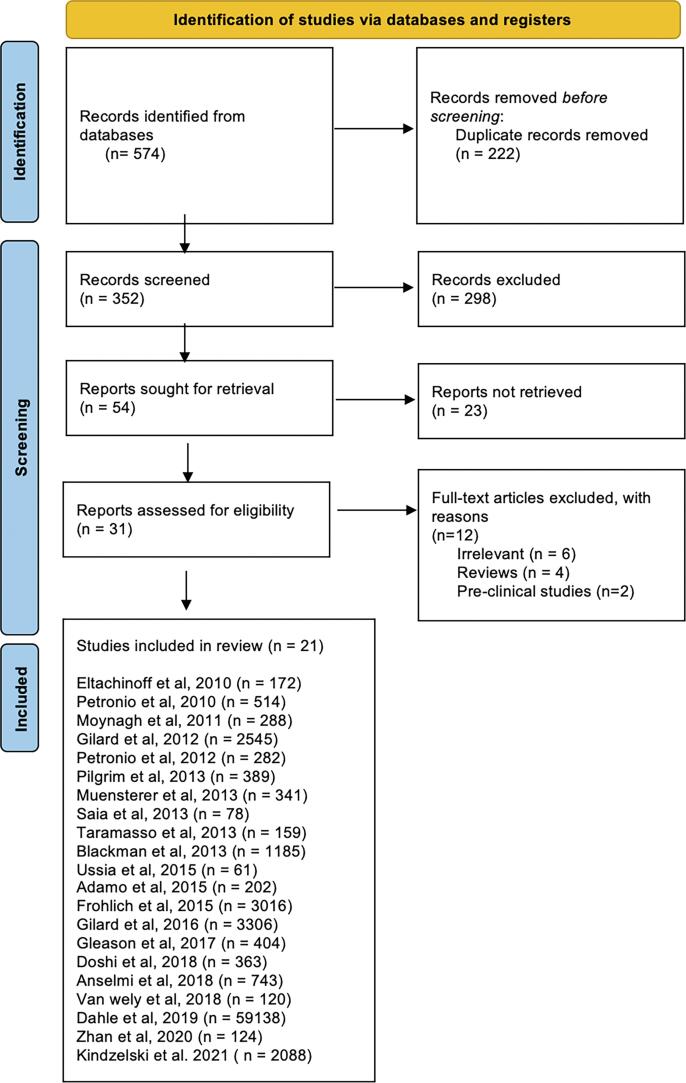
PRISMA Flow Diagram of Study Selection.

**Fig. 2:**
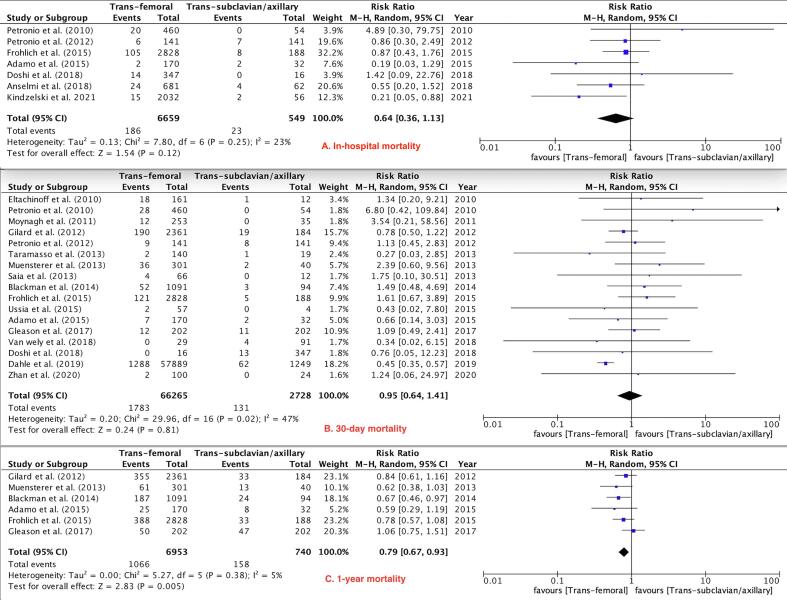
Mortality Outcomes.

### 2.4 Outcome measures

The co-primary outcomes for study selection were in-hospital, 30-day, and 1-year all-cause mortality, major vascular complication, major bleeding and stroke. Peri-procedural myocardial infarction (MI), cardiac tamponade, pacemaker placement, conversion to open surgery, acute kidney injury (AKI), procedure success, procedure time and fluoroscopy time were secondary outcomes. Outcome definitions were as determined in each individual study. Most outcomes were assessed according to Valve Academic Research Consortium (VARC) definitions.[18].

### 2.5 Statistical analysis

For dichotomous outcomes, risk ratios (RRs) with 95 % confidence intervals (CIs) were calculated from the available data, and trial-specific RRs were combined using the DerSimonian and Laird random effects model with the estimate of heterogeneity taken from the Mantel–Haenszel model. We used I^2^ statistics to measure heterogeneity among the included trials. A value of 0 % indicated no observed heterogeneity, and I^2^ values of 25 %, 50 %, and 75 % were considered to represent low, moderate, and high heterogeneity, respectively. Analyses were performed using Review Manager (RevMan) Version 5.3 (The Nordic Cochrane Centre, The Cochrane Collaboration, Copenhagen, Denmark).

## 3 Results

### 3.1 Search results

Fig. 1 displays the flow diagram for study search and selection. A total of 21 studies [12], [13], [19], [20], [21], [22], [23], [24], [25], [26], [27], [28], [29], [30], [31], [32], [33], [34], [35], [36] including 75,995 unique patients who underwent TAVR (73,203 TF and 2,792 TSc/TAx) were included in the *meta*-analysis. All of the included studies were observational studies. The characteristics of the included studies and the patients’ clinical profiles and demographic features are presented in Tables 1 **and 2**, respectively.

**Table 1:** Study characteristics of the included investigations. TF = transfemoral, TSc = *trans*-subclavian, TA = transaxillary.

Study	Year	Region/ Country	Study Design	Enrollment Period	Number of patients	Type of access		Type of valve	Follow-up duration
						TF	TSc/TA		
Eltachinoff et al	2010	France	Prospective observational, multicenter	02/2009–06/2009	173	161	12	Edwards SAPIENTM or CoreValveTM	1-month
Petronio et al	2010	Italy	Prospective observational, multicenter	06/2007–07/2009	514	460	54	CoreValve	In-hospital, 6-month
Moynagh et al	2011	UK and Ireland	Retrospective observational, multicenter	04/2007–04/2010	288	253	35	CoreValve	1-month
Gilard et al	2012	France	Prospective observational, multicenter	01/2010–10/2011	2545	2361	184	. Edwards SAPIEN and Medtronic CoreValve devices	1-month, 1-year
Petronio et al	2012	Italy	Prospective observational, multicenter	06/2007–03/2011	282	141	141	CoreValve	Procedural results, in-hospital, 1-month, 2-years
Muensterer et al	2013	Germany	Prospective observational, single center	06/2007–02/2011	341	301	40	CoreValve	1-month, 6-months, 1-year
Saia et al	2013	Italy	Prospective observational, single center	2008–11/2010	78	66	12	Medtronic CoreValve, Edwards-Sapiens, and Sapiens-XT	1-month
Taramasso et al	2013	Italy	Prospective observational, single center	11/2007–06/2010	159	140	19	SAPIEN XT, third generation CoreValve	1-month, in-hospital
Blackman et al	2013	UK	Prospective observational, multicenter	01/2007–12/2010	1185	1091	94	SAPIEN and CoreValve	1-month, 1-year, 2-years
Ussia et al	2015	Italy	Prospective observational, single center	01/2012–07/2013	61	57	4	CoreValve	In-hospital, 1-month
Adamo et al	2015	Italy	Prospective observational, single center	09/2007–03/2014	202	170	32	Medtronic CoreValve	1-month, 1-year
Frohlich et al	2015	UK	Retrospective observational, multicenter	01/2007–12/2012	3016	2828	188	CoreValve and Edwards SAPIEN	In-hospital, 1-month, 1-year
Gilard et al	2016	France	Prospective observational, multicenter	01/2010–01/2012	3306	3064	242	Medtronic CoreValve and Edwards SAPIEN	1-month, 6-months, and 1, 2, 3, 4, 5 years (mean 3.8).
Gleason et al	2017	USA	Prospective observational, single center	N/A	404	202	202	CoreValve	1-month, 1-year
Doshi et al	2018	UK	Prospective observational, single center	12/2008–10/2016	363	347	16	Edwards SAPIEN XT, Edwards SAPIEN 3, Lotus valve, CoreValve, Evolute R	In-hospital, 1-month
Anselmi et al	2018	France	Prospective observational, single center	01/2002–12/2016	743	681	62	CoreValve, Edwards XT, Edwards SAPIEN 3, CoreValve EvolutR, Edwards Centera, Saint JudePortico-CoreValve	In-hospital, 1-month
Van wely et al	2018	Netherlands	Prospective observational, single center	09/2015–07/2017	120	29	91	Portico or CoreValve	1-month
Dahle et al	2019	USA	Retrospective observational, multicenter	06/2015–02/2018	59,138	57,889	1249	Evolut	Procedural results, in-hospital, 1-month, 2 years
Zhan et al	2020	USA	Retrospective observational, single center	08/2015–06/2019	124	100	24	Edwards SAPIEN 3	1-month
Kindzelski et al	2021	USA	Retrospective observational, single center	01/2006–01/2019	2088	2032	56	SAPIEN 3 and CoreValve	In-hospital, 2–5 years

**Table 2:** Basic characteristics of the included studies patients.

Studies	Access site	Baseline characteristics																
		Male (%)	Age (mean ± SD) or median	STS score	Logisitic Euroscore	DM (%)	HTN (%)	Prior MI (%)	Prior PCI (%)	Prior CABG (%)	Prior stroke (%)	CAD (%)	AF (%)	Carotid stenosis (%)	PAD (%)	Porcelain aorta (%)	NYHA class III/IV (%)	Previous pacemaker (%)
Eltachinoff et al, 2010	Transfemoral	46.6	82.9 ± 6.6	19.4 ± 13.1	25.2 ± 11.3	28.6	68.3	26	N/A	23.6	9.9	38.5	N/A	N/A	N/A	N/A	76.4	17.4
	Subclavian	50	75.5 ± 11	21 ± 17.2	24.6 ± 14.6	8.3	50	25	N/A	33.3	8.3	50	N/A	N/A	N/A	N/A	50	25
Petronio et al, 2010	Transfemoral	41.3	83(78–86)	N/A	19.4(12.5–29.8)	27.4	74.8	20.7	27	16.5	7	48.7	N/A	11.1	15	12.4	69.7	10.4
	Subclavian	66.7	83(80–86)	N/A	25.3(15.1–36.6)	20.4	74.1	33.3	46.3	14.8	14.8	64.8	N/A	20.4	55.6	16.7	54.7	3.7
Moynagh et al, 2011	Transfemoral	N/A	81.7 ± 6.4	N/A	19.1 ± 12.3	N/A	N/A	16.2	24.1	N/A	N/A	58.5	N/A	N/A	21.3	N/A	N/A	N/A
	Subclavian	N/A	80.6 ± 4.9	N/A	25 ± 14.7	N/A	N/A	34.3	34.3	N/A	N/A	74.2	N/A	N/A	74.2	N/A	N/A	N/A
Gilard et al, 2012	Transfemoral	47.4	83 ± 7.2	14.5 ± 11.9	21.2 ± 14.7	N/A	N/A	14.5	N/A	15.2	N/A	44.4	27.9	N/A	12.5	N/A	77.8	N/A
	Subclavian	71.2	82.2 ± 6.7	16.6 ± 13.4	20.3 ± 14.7	N/A	N/A	18.5	N/A	24.2	N/A	58.4	31.5	N/A	41.6	N/A	71.4	N/A
Petronio et al, 2012	Transfemoral	57.7	83(78.6–86.1)	N/A	23.3(15.8–33.6)	N/A	N/A	N/A	37.6	N/A	9.2	48.9	N/A	N/A	20.6	N/A	68	N/A
	Subclavian	61	83(78.9–87)	N/A	23.7(13.5–32.7)	N/A	N/A	N/A	48.2	N/A	12.8	58.9	N/A	N/A	85.1	N/A	72.3	N/A
Muensterer et al, 2013	Transfemoral	44.9	80.2 ± 7.0	5.9 ± 4.1	19.2 ± 12.8	N/A	N/A	N/A	N/A	N/A	N/A	52.2	N/A	N/A	14	3.3	95.3	N/A
	Subclavian	57.5	79.5 ± 8.5	6.6 ± 5.6	21.5 ± 12.2	N/A	N/A	N/A	N/A	N/A	N/A	60	N/A	N/A	42.5	5	100	N/A
Saia et al, 2013	Transfemoral	83.7 ± 5.3	N/A	N/A	N/A	N/A	N/A	N/A	N/A	N/A	N/A	N/A	N/A	N/A	N/A	N/A	N/A	N/A
	Subclavian		N/A	N/A	N/A	N/A	N/A	N/A	N/A	N/A	N/A	N/A	N/A	N/A	N/A	N/A	N/A	N/A
Taramasso et al, 2013	Transfemoral	53.1	79.8 ± 6.5	20.6 ± 12	26.7 ± 15.8	18.6	N/A	22.1	N/A	N/A	N/A	N/A	N/A	N/A	18.6	12.1	70	N/A
	Transaxillary	73.7	79.7 ± 5.5	22.3 ± 13.2	28.6 ± 14.3	26.3	N/A	36.8	N/A	N/A	N/A	N/A	N/A	N/A	63.1	36.8	60	N/A
Blackman et al, 2013	Transfemoral	52.5	81.7 ± 7.5	N/A	18.6 ± 13.3	22	N/A	21.9	N/A	N/A	N/A	44.3	N/A	N/A	17.6	N/A	N/A	N/A
	Subclavian	68.1	82 ± 6.5	N/A	25.9 ± 16.9	23	N/A	25.3	N/A	N/A	N/A	51.1	N/A	N/A	55.3	N/A	N/A	N/A
Ussia et al, 2015	Transfemoral	N/A	N/A	N/A	N/A	N/A	N/A	N/A	N/A	N/A	N/A	N/A	N/A	N/A	N/A	N/A	N/A	N/A
	Transaxillary	N/A	N/A	N/A	N/A	N/A	N/A	N/A	N/A	N/A	N/A	N/A	N/A	N/A	N/A	N/A	N/A	N/A
Adamo et al, 2015	Transfemoral	62	83 ± 7	6.7(4.7–11.2)	18(11–27)	28	71	17	24	18	N/A	43	22	N/A	11	N/A	75	N/A
	Transaxillary	44	82 ± 6	8.3(5.6–14)	26(20–33)	28	72	16	41	12	N/A	53	47	N/A	66	N/A	75	N/A
Frohlich et al, 2015	Transfemoral	51	83(77–87)	N/A	17(11–26)	23	N/A	22	21	N/A	N/A	42	21	N/A	N/A	N/A	N/A	N/A
	Subclavian	65	83(78–86)	N/A	22(14–34)	23	N/A	28	24	N/A	N/A	51	17	N/A	N/A	N/A	N/A	N/A
Gilard et al, 2016	Transfemoral	N/A	N/A	N/A	N/A	N/A	N/A	N/A	N/A	N/A	N/A	N/A	N/A	N/A	N/A	N/A	N/A	N/A
	Subclavian	N/A	N/A	N/A	N/A	N/A	N/A	N/A	N/A	N/A	N/A	N/A	N/A	N/A	N/A	N/A	N/A	N/A
Gleason et al, 2017	Transfemoral	58.9	80.2 ± 9.7	9.8 ± 5.5	19.4	43.1	94.6	31.2	40.1	N/A	10.4	83.7	52.5	N/A	57.9	N/A	89.6	N/A
	Subclavian/axillary	63.9	80.8 ± 8.1	9.7 ± 5.9	20.7	43.1	91.6	31.7	40.1	N/A	9.9	81.7	48.5	N/A	60.4	N/A	88.6	N/A
Anselmi et al, 2018	Transfemoral	52	81.58	N/A	N/A	N/A	N/A	N/A	N/A	N/A	N/A	N/A	N/A	N/A	N/A	N/A	N/A	N/A
	Subclavian	61	79.38	N/A	N/A	N/A	N/A	N/A	N/A	N/A	N/A	N/A	N/A	N/A	N/A	N/A	N/A	N/A
Doshi et al, 2018	Transfemoral	55	83(78–86)	N/A	14(10–24)	31	N/A	22	22	N/A	N/A	N/A	25	N/A	21	N/A	N/A	20
	Transaxillary	75	78(72–84)	N/A	19(15–24)	38	N/A	44	38	N/A	N/A	N/A	38	N/A	81	N/A	N/A	31
Van wely et al, 2018	Transfemoral	N/A	82(78–85)	N/A	18.5 ± 10	N/A	N/A	N/A	N/A	N/A	N/A	N/A	N/A	N/A	N/A	N/A	N/A	N/A
	Subclavian	N/A	80(75–83)	N/A	13.9 ± 9.5	N/A	N/A	N/A	N/A	N/A	N/A	N/A	N/A	N/A	N/A	N/A	N/A	N/A
Dahle et al, 2019	Transfemoral	55.4	80.8 ± 8.3	6.6 ± 4.6	N/A	38.2	90.5	N/A	33.2	21	11.2	N/A	N/A	23.8	25.3	3.3	76.1	N/A
	Transaxillary	58.9	78.9 ± 8.7	7.7 ± 5.8	N/A	42.4	92.9	N/A	37.7	25.1	12.2	N/A	N/A	42.5	67	7.6	80	N/A
Zhan et al. 2020	Transfemoral	48	80.5 ± 7.6	7.3 ± 5.2	N/A	38	86	N/A	19	16	11	76	35	N/A	N/A	N/A	90	11
	Transaxillary	41.7	82.9 ± 8.8	11.3 ± 7.6	N/A	62.5	83.3	N/A	45.8	16.7	20.8	79.2	25	N/A	N/A	N/A	91.7	0
Kindzelski et al. 2021	Transfemoral	58	81 ± 9.5	5.5(3.0–11)	N/A	37	89	22	N/A	N/A	13	N/A	38	N/A	23			4
	Transaxillary	57	80 ± 7.8	7.0(3.3–11)	N/A	24	93	21	N/A	N/A	20	N/A	38	N/A	61			7.3

### 3.2 Outcomes

**Fig. 3:**
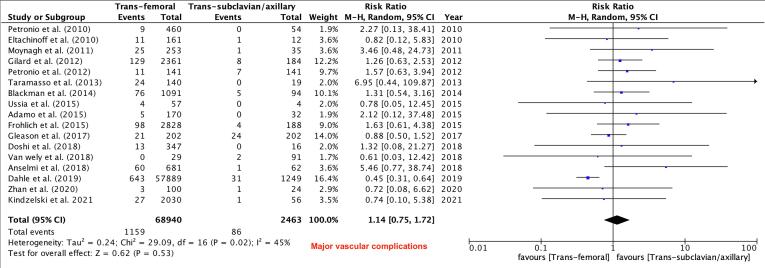
Major Vascular Complications.

**Fig. 4:**
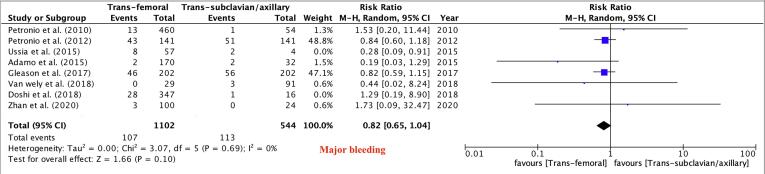
Major Bleeding.

**Fig. 5:**
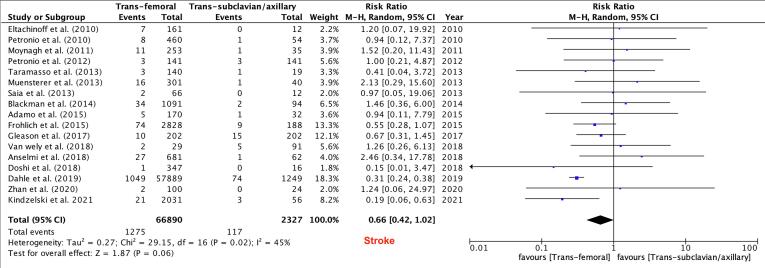
Stroke.

Primary Outcomes: There was no difference between the TF and TSx/TAx groups in terms of in-hospital and 30-day mortality (RR 0.64, 95 % CI 0.36–1.13, P = 0.12) and (RR 0.95, 95 % CI 0.64–1.41, P = 0.81) respectively, while 1-year mortality was lower in the TF TAVR group (RR 0.79, 95 % CI 0.67–0.93, P = 0.005, Fig. 2). There were no differences between the two groups in the risk of major vascular complications (RR 1.14, 95 % CI 0.75–1.72, P = 0.53, Fig. 3),major bleeding (RR 0.82, 95 % CI 0.65–1.03, P = 0.09, Fig. 4) and stroke rates (RR 0.66, 95 % CI 0.42–1.02, P = 0.06, Fig. 5).

**Fig. 6:**
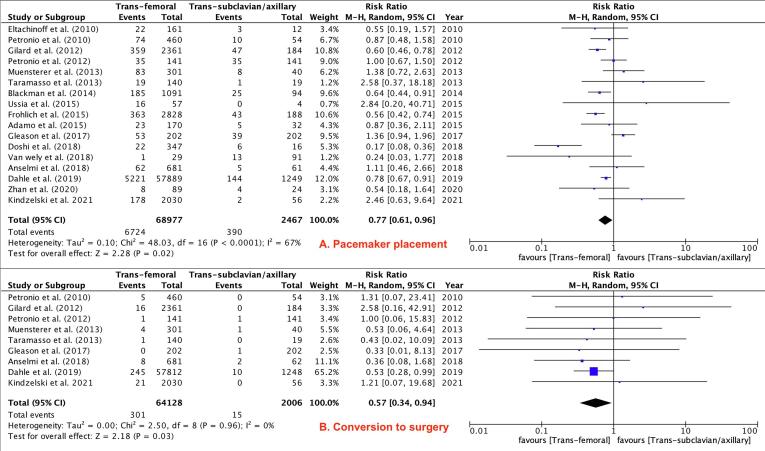
Pacemaker Placement and Conversion to Surgery.

**Supplementary figure 3:**
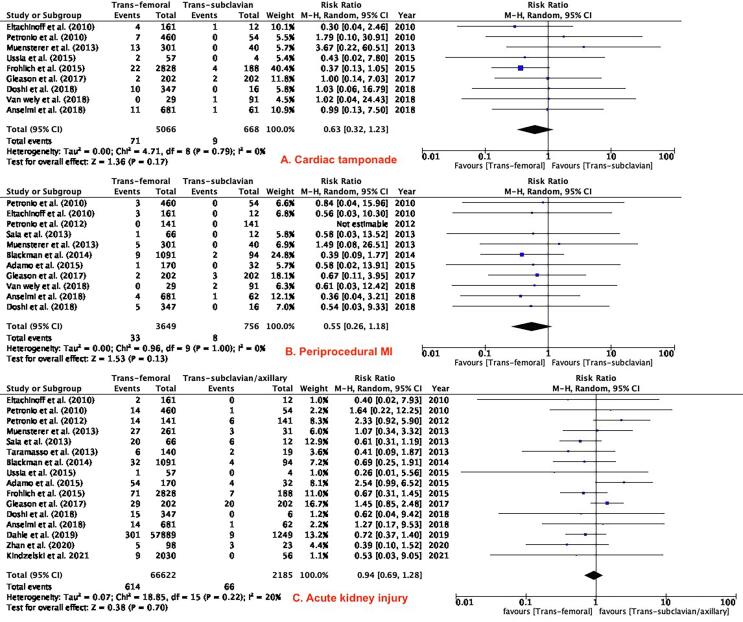


**Supplementary figure 4:**
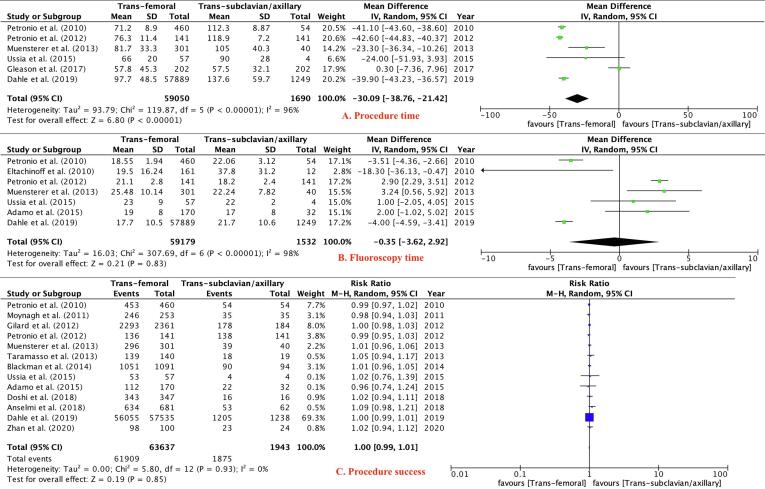

Secondary Outcomes: TF TAVR was associated with less pacemaker placement (RR 0.77; 95 % CI 0.61–0.96, P = 0.02, Figure 6**-A**) and less conversion to open surgery (RR 0.57; 95 % CI 0.34–0.94, P = 0.03, Figure 6-B) when compared to TSc/TAx TAVR. There were no differences between the two groups in rates of cardiac tamponade (RR 0.63; 95 % CI 0.32–1.23; P = 0.17, **Supplemental** Fig. 3**-A**), periprocedural MI (RR 0.55; 95 % CI 0.26–1.18; P = 0.13, Supplemental Fig. 3-B), and AKI (RR 0.94, 95 % CI 0.69–1.28, P = 0.70, **Supplemental** Fig. 3**-C**). When compared to TSc/TAx TAVR, TF TAVR was associated with shorter procedure time (RR [-30.09], 95 % CI [-38.76, −21.42], P < 0.00001, **Supplemental** Fig. 4**-A**) but no difference in the fluoroscopy time (RR [-0.35], 95 % CI [- 3.62, 2.92], P = 0.83, **Supplemental** Fig. 4**-B**). Procedural success rates were similar in both groups (RR 1.00, 95 % CI 0.99–1.01, P = 0.85, **Supplemental** Fig. 4**-C**).

With respect to clinical outcomes, there was no significant heterogeneity for in-hospital mortality (P = 0.27, I^2^ = 0 %), 1-year mortality (P = 0.005, I^2^ = 5 %), major bleeding (P = 0.09, I^2^ = 0 %), periprocedural MI (P = 0.13, I^2^ = 0 %), cardiac tamponade (P = 0.17, I^2^ = 0 %), conversion to open surgery (P = 0.02, I^2^ = 0 %), or procedure success (P = 0.85, I^2^ = 0 %). There was low to moderate heterogenicity for 30-day mortality (P = 0.81, I^2^ = 47 %), stroke (P = 0.2, I^2^ = 46 %), major vascular complications (P = 0.48, I^2^ = 48 %), and AKI (P = 0.73, I^2^ = 25 %). The heterogenicity was considerable for pacemaker placement (P = 0.01, I^2^ = 67 %), fluoroscopy time (P = 0.05, I^2^ = 98 %), and procedure time (P < 0.00001, I^2^ = 96 %). Overall, heterogeneity was low and there was no evidence of publication bias on visual inspection of funnel plot **(Supplemental** Fig. 1, Fig. 2**)**.

## 4 Discussion

Our analysis of 21 studies including more than 75,000 patients showed: (1) There was no significant difference in in-hospital, and 30-day mortality between patients undergoing TF vs TSc/TAx TAVR, while 1-year mortality was lower in the TF group. (2) There were no significant differences between the two groups in the risks of major bleeding and major vascular complications. (3) Rate of pacemaker placement were significantly less in the TF access group. (4) Stroke did not differ significantly between the groups. (5) Cardiac tamponade, *peri*-procedural MI, and AKI, did not differ significantly between the groups. (6) The procedure time was noted to be lower in the TF group, with no significant difference in fluoroscopy time.

### 4.1 Mortality

Ruge and colleagues [37] were the first to describe successful TSc/TAx-TAVR in a patient with aortoiliac occlusive disease and concomitant left subclavian arterial stenosis (the right SCA was accessed). Use of the left SCA was later described by Asgar and colleagues [38] in 2009 after they treated a woman with severe aortic stenosis and very small iliofemoral arteries. Data from previous studies support the TSc/TAx as the preferred non-TF route due to several advantages.[34], [39], [40] Gleason et al[27] compared a cohort of TSc patients to TF patients within the CoreValve US Pivotal Trial and Continued Access Study, and reported TSc patients, demonstrated no significant differences in outcomes, with 30-day and 1-year mortality rates equivalent to TF procedures. That aligns with the results of our pooled analysis with respect to 30-day mortality, while contrasting with our study with respect to 1 year mortality, which was noted to be higher in patients with TSc/TAx approach. A possible explanation may relate to the higher rate of comorbidities seen in TSc and TAx groups than the TAVI procedure itself. TSc/TAx patients have increased risk, reflected by the higher Logistic EuroSCORE as seen in table 2, which may explain the worse late survival. Iliofemoral disease is the most common reason that makes iliofemoral access undesirable. Peripheral vascular disease (PVD) especially iliofemoral disease is frequently seen in patient being referred for TSc/TAx TAVR with prevalence ranging from 43 % to 60 % and.[41] It is a well-known fact that PAD is an independent predictor of long term mortality and stroke in these patients.[41], [42] Table 2 shows higher prevalence of baseline PVD in patients undergoing TSc/TAx -TAVR than TF approach which is in accordance with prior studies.

### 4.2 Bleeding and vascular complications

Percutaneous or surgical cut down access for TSc/TAx TAVR were used in the included studies. However, major vascular complication rates appear not to be significantly different between the two groups. This supports the notion that the TSc/TAx approach may be the preferred alternative-access option in the current era of newer-generation devices. Pooled results deriving from unadjusted data in our *meta*-analysis found no difference in the risk of major bleeding in both groups although was a non-significant trend towards decrease in the risk of major bleeding the TF TAVR group. This is in line with a previous analysis [43] that used adjusted data and showed no statistically difference in the risk of bleeding during transcarotid/transsubclavian TAVR in comparison with transfemoral TAVR. Another report from the FRANCE registry who grouped TC and subclavian/axillary TAVR (1,616 patients) reported similar outcomes compared to TF in the term of major bleeding.[44] In our *meta*-analysis we compared transsubclavian/transaxillary TAVR without transcarotid (TC) TAVR group to the femoral TAVR.

### 4.3 Pacemaker rates

Conduction dysfunction originating from the mechanical injury due to the anatomical interaction between the valve prosthesis and the atrioventricular node and bundle of His are the implicated causes requiring pacemaker implantation. [45] In our metanalysis, we found that the rate of pacemaker placement was surprisingly lower in TF approach compared to TSc/TAx approach. Moreover, there was no differences in procedural complications such as MI, AKI, and cardiac tamponade. These findings are consistent with Italian CoreValve Registry data that showed comparable procedural and 2-year results after TSc and TF approaches.[40] Our *meta*-analysis showed that the TSc/TAx approach had a longer procedure time when compared to TF approach, which exposes the AV node area to longer manipulation time that could lead to AV nodal dysfunction.

### 4.4 Stroke

Previous studies have reported conflicting data in regards to *peri*-procedural stroke events. Dahle et al reported higher stroke rates in TAx approach, which may be partially related to an increased risk of access site trauma with TSc/TAx approach.[38], [46] providing nidus for thrombus formation with subsequent embolization or embolization of an atheromatous plaque located in the subclavian artery. This makes potentially relevant the use of embolic protection during TSc/TAx approach.[47]. However, other included studies as well as our aggregate analysis reported no significant difference in post-procedural stroke with either access.

### 4.5 Procedure and fluoroscopy time

The procedure time was reported in 6 studies and fluoroscopy time in 7 studies. Petronio et al.[40] found that the overall procedural time was longer in the TSc group compared to the TF (120 vs 75 min, p < 0.0001), however the fluoroscopy time was similar (18 vs 21 min, p = 0.15). Muensterer et al.[13] also failed to demonstrate a significant difference in fluoroscopy time between the TSc and TF groups (22.24 vs 25.48 min, p = 0.053) which is aligned with our pooled analysis that showed comparable fluoroscopy time in both groups, however the procedural time was significantly longer in the TSc group (105 vs 82 min, p = 0.001). Dahle et al.[28] found that the mean total fluoroscopy time and procedure time were slightly longer in the TSc group compared to TF groups (21.7 vs 17.7 min and 137.6 vs 97.7 min, respectively). Our *meta*-analysis showed no difference in the fluoroscopy time while procedure time was shorter in the TF TAVR group. Since the fluoroscopy time is similar in both groups our findings could be explained by the longer surgical vascular access and wound closure required in the TSc/TAx group, in addition to that fact that most operators will have potentially more expertise with the TF approach.

A previous *meta*-analysis by Zhan et al.[48] of 5 studies comparing the TF and TSc/TAx and another network *meta*-analysis [49] comparing several access sites demonstrated a lower but statistically non-significant 1-year mortality with the TF group, while our study showed statistically significant lower 1 year mortality in the TF group. Our present *meta*-analysis included 21 studies with subclavian/transaxillary and 75,995 patients. We also found a lower rate of new pacemaker placement in TF group compared TSc/TAx, which is different compared to the previous *meta*-analyses who reported comparable risk of new pacemaker between the two groups. Furthermore, we evaluated other outcomes such as procedure time and fluoroscopy time, which were not part of the outcomes of interest in the prior *meta*-analysis, thus, our *meta*-analysis adds methodological rigor and novel findings to the literature.

### 4.6 Limitations

Our study has several important limitations. In this study, the data analyzed were from observational studies and not randomized trials comparing TF and TSc/TAx access. There is intrinsic heterogeneity between different studies in terms of representation of baseline data, study design, and outcome measures. Only one study included in this *meta*-analysis was propensity-matched with similar patient demographics, other studies exhibited major differences in baseline characteristics between the TF and TSc/TAx cohorts. There is a possibility of publication bias among the outcomes where significant asymmetry was observed. Moreover, data included in our analysis represents a conglomerate of both self-expandable and balloon-expandable prostheses making it unattainable to carry out a head to head comparison between such devices. The data of pre-dilation or direct implantation were not available in the studies as well. Lastly, the quality of this *meta*-analysis reflects the quality of individual studies. Nevertheless, our *meta*-analysis is strengthened by inclusion of a large number of real world studies (total 21) and therefore, is the most current and comprehensive *meta*-analysis on this important clinical issue.

## 5 Conclusion

In patients undergoing TAVR, TF access is associated with significantly lower 1-year mortality compared to TSc/TAx access, while there were no differences in major vascular complications, major bleeding or stroke. While TF is the preferred approach for TAVR, TSc/TAx appears to be a safe alternative approach. Future studies should confirm these findings, preferably in a randomized setting.

## Declaration of Competing Interest

The authors declare that they have no known competing financial interests or personal relationships that could have appeared to influence the work reported in this paper.

## Figures and Tables

**Supplementary figure 2:**
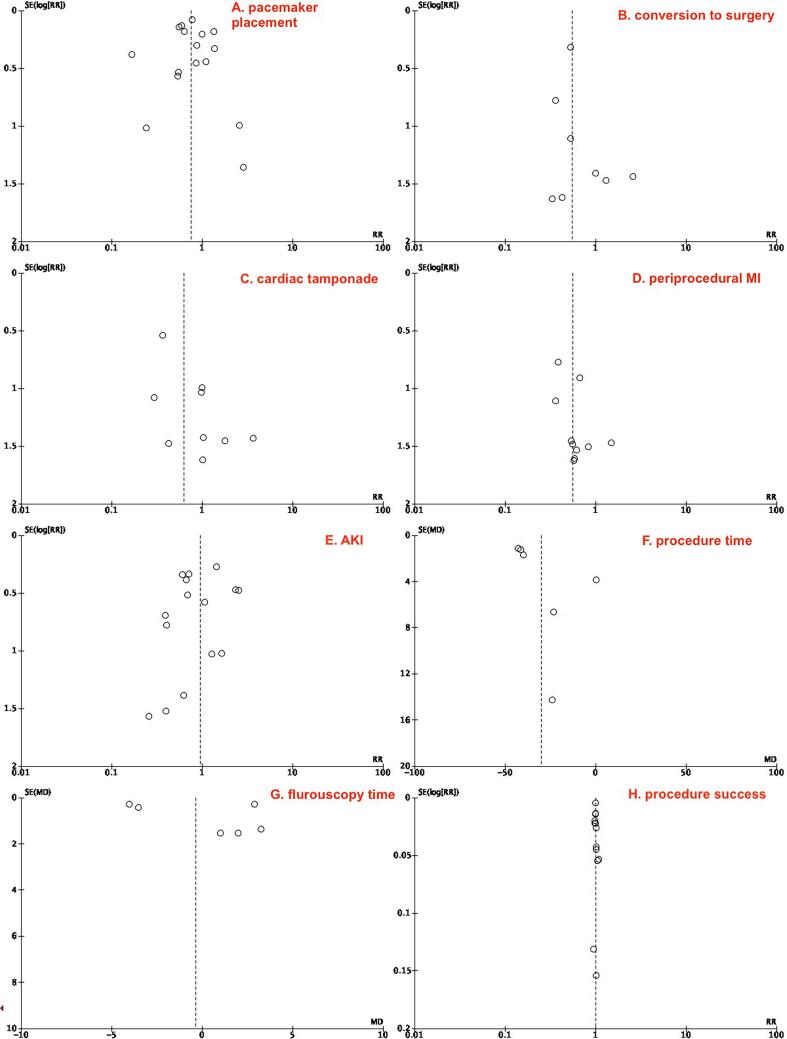

