# Supplementary material for: Comparison of Transfemoral versus Transsubclavian/Transaxillary access for transcatheter aortic valve replacement: A systematic review and meta-analysis
Source: Int J Cardiol Heart Vasc. 2022 Dec 1;43:101156. doi: 10.1016/j.ijcha.2022.101156 (PMC9718962; doi:10.1016/j.ijcha.2022.101156)
Supplement: Supplementary data 1 [file mmc1.docx]

Supplementary Table 1: Search Strategy

aortic stenosis”

| Pubmed | | | | |
| --- | --- | --- | --- | --- |
| No. | Search terms | | | Number of items |
| 1 | **Aortic stenosis** | "aortic valve stenosis"[MeSH Terms] OR ("aortic"[All Fields] AND "valve"[All Fields] AND "stenosis"[All Fields]) OR "aortic valve stenosis"[All Fields] OR ("aortic"[All Fields] AND "stenosis"[All Fields]) OR "aortic stenosis"[All Fields] | | 66,884 |
| 2 | **(transcatheter aortic valve replacement) OR (TAVR)) OR (TAVI)** | "transcatheter aortic valve replacement"[MeSH Terms] OR ("transcatheter"[All Fields] AND "aortic"[All Fields] AND "valve"[All Fields] AND "replacement"[All Fields]) OR "transcatheter aortic valve replacement"[All Fields] OR "TAVR"[All Fields] OR "TAVI"[All Fields] | | 16,119 |
| 3 | **(subclavian artery access) OR (axillary artery access)) OR (femoral artery access)** | ("subclavian artery"[MeSH Terms] OR ("subclavian"[All Fields] AND "artery"[All Fields]) OR "subclavian artery"[All Fields]) AND ("access"[All Fields] OR "accessed"[All Fields] OR "accesses"[All Fields] OR "accessibilities"[All Fields] OR "accessibility"[All Fields] OR "accessible"[All Fields] OR "accessing"[All Fields])) OR (("axillary artery"[MeSH Terms] OR ("axillary"[All Fields] AND "artery"[All Fields]) OR "axillary artery"[All Fields]) AND ("access"[All Fields] OR "accessed"[All Fields] OR "accesses"[All Fields] OR "accessibilities"[All Fields] OR "accessibility"[All Fields] OR "accessible"[All Fields] OR "accessing"[All Fields])) OR (("femoral artery"[MeSH Terms] OR ("femoral"[All Fields] AND "artery"[All Fields]) OR "femoral artery"[All Fields]) AND ("access"[All Fields] OR "accessed"[All Fields] OR "accesses"[All Fields] OR "accessibilities"[All Fields] OR "accessibility"[All Fields] OR "accessible"[All Fields] OR "accessing"[All Fields]) | | 5,545 |
| 4 | **Combined search** | #1 AND #2 AND #3 AND #4 | | 412 |
| Cochrane Library | | | | |
| 1 | Severe aortic stenosis | | | 956 |
| 2 | Transcatheter aortic valve replacement | | | 823 |
| 3 | Subclavian artery access | | | 85 |
| 4 | Axillary artery access | | | 71 |
| 5 | Femoral artery access | | | 716 |
|  | Combined search | | #1 and #2 and #5 Or #3 Or #4 | 148 |
| ClinicalTrials.gov | | | | |
| 1 | Aortic valve stenosis, transcatheter aortic valve replacement, femoral artery access | | | 14 |
