# Supplementary material for: Comparison of Transfemoral versus Transsubclavian/Transaxillary access for transcatheter aortic valve replacement: A systematic review and meta-analysis
Source: Int J Cardiol Heart Vasc. 2022 Dec 1;43:101156. doi: 10.1016/j.ijcha.2022.101156 (PMC9718962; doi:10.1016/j.ijcha.2022.101156)
Supplement: Supplementary data 2 [file mmc2.docx]

| Study | Selection | | | | Comparability (Confounding) | Outcome | | | Total |
| --- | --- | --- | --- | --- | --- | --- | --- | --- | --- |
|  | Repres-ntative of exposed cohort | Selection of the non-exposed cohort | Ascertain-ment of exposure | Endpoint not present at start |  | Asse-ssment of Outcome | Follow-up duration | Adequ-acy follow-up |  |
| Eltachinoff et al | ⁕ | * | ⁕ | ⁕ | ** | ⁕ | ⁕ | ⁕ | 9 |
| Petronio et al | ⁕ | * | ⁕ | ⁕ | ** | ⁕ | ⁕ | ⁕ | 9 |
| Moynagh et al | ⁕ | * | ⁕ | ⁕ | * | ⁕ | ⁕ | * | 8 |
| Gilard et al | ⁕ | * | ⁕ | ⁕ | ** | ⁕ | ⁕ | ⁕ | 9 |
| Petronio et al | ⁕ | * | ⁕ | ⁕ | ** | ⁕ | ⁕ | ⁕ | 9 |
| Muensterer et al | ⁕ | * | ⁕ | ⁕ | ** | ⁕ | ⁕ | * | 9 |
| Saia et al | ⁕ | * | ⁕ | ⁕ | ** | ⁕ | ⁕ | ⁕ | 9 |
| Taramasso et al | ⁕ | * | ⁕ | ⁕ | ** | ⁕ | ⁕ | ⁕ | 9 |
| Blackman et al | ⁕ | * | ⁕ | ⁕ | ** | ⁕ | ⁕ | ⁕ | 9 |
| Ussia et al | ⁕ | * | ⁕ | ⁕ | ** | ⁕ | ⁕ | ⁕ | 9 |
| Adamo et al | ⁕ | * | ⁕ | ⁕ | ** | ⁕ | ⁕ | ⁕ | 9 |
| Frohlich et al | ⁕ | * | ⁕ | ⁕ | ⁕⁕ | ⁕ | ⁕ | ⁕ | 9 |
| Gilard et al | ⁕ | ⁕ | ⁕ | ⁕ | ⁕⁕ | ⁕ | ⁕ | ⁕ | 9 |
| Gleason et al | ⁕ | ⁕ | ⁕ | ⁕ | ⁕⁕ | ⁕ | ⁕ | ⁕ | 9 |
| Doshi et al | **⁕** | ⁕ |  | ⁕ | ⁕⁕ | ⁕ | ⁕ | ⁕ | 8 |
| Anselmi et al | **⁕** | ⁕ | ⁕ | ⁕ | ⁕⁕ | ⁕ | ⁕ | ⁕ | 9 |
| Van wely et al | **⁕** | ⁕ |  | ⁕ | ⁕⁕ | ⁕ | ⁕ | ⁕ | 8 |
| Dahle et al | **⁕** | ⁕ | ⁕ | ⁕ | ⁕ | ⁕ | ⁕ | ⁕ | 8 |
| Zhan et al | **⁕** | ⁕ | ⁕ | ⁕ | ⁕⁕ | ⁕ | ⁕ | ⁕ | 9 |
| Kindzelski et al | **⁕** | ⁕ | ⁕ | ⁕ | ⁕ | ⁕ | ⁕ | ⁕ | 8 |
| Pilgrim et al | **⁕** | ⁕ | ⁕ | ⁕ | ⁕⁕ | ⁕ | ⁕ | ⁕ | 9 |

**Supplementary Table.** The Newcastle-Ottawa scale assessing the quality of included studies. Notes: The Newcastle-Ottawa scale uses a star system (0 to 9) to evaluate included studies on 3 domains: selection, comparability, and outcomes. Star (*) = item presents. Maximum 1 star (*) for selection and outcome components and 2 stars (**) for comparability components. Higher scores represent higher study quality.
